# Postinjury Neuroplasticity in Central Neural Networks

**DOI:** 10.1155/2015/857085

**Published:** 2015-10-18

**Authors:** Bae Hwan Lee, Tae-Hong Lim, Young Wook Yoon, Midori A. Yenari, Yong Jeong

**Affiliations:** ^1^Department of Physiology, Brain Korea 21 PLUS Project for Medical Science, Yonsei University College of Medicine, Seoul 120-752, Republic of Korea; ^2^Department of Biomedical Engineering, Seamans Center for the Engineering Arts and Sciences, College of Engineering, University of Iowa, Iowa City, IA 52242, USA; ^3^Department of Physiology, Korea University College of Medicine, Seoul 136-705, Republic of Korea; ^4^Department of Neurology, University of California, San Francisco, and San Francisco Veterans Affairs Medical Center, San Francisco, CA 94121, USA; ^5^Department of Bio and Brain Engineering, Korea Advanced Institute of Science and Technology, Daejeon 305-701, Republic of Korea

Traumatic insult, ischemia, or degenerative disorder can damage neuronal cell bodies, axons, or synapses in the complex circuitry of the central nervous system (CNS). Peripheral as well as central injuries induce tremendous changes at the molecular, cellular, and system levels in the spinal cord and brain, which lead to devastating losses of functions including sensory, motor, and higher-order functions. Symptoms related to functional deficits depend on the site and extent of injury. Functional deficits caused by neural injury reflect the disruption of the intricate central neural circuits.

After injury, neuronal death or dysfunction occurs in affected areas. Sometimes, however, certain changes may occur in the adjacent and/or remote areas of the nervous system. The CNS possesses plasticity to respond to injury of either the peripheral nerves, the spinal cord, or the brain. Due to neural plasticity, lost functions may be restored. The plastic changes are often maladaptive as shown in neuropathic pain and are exacerbated hypersensitivity.

Although the regeneration capacity of central neurons is limited, extensive changes can occur in the CNS circuitry after injury, reflecting neuroplasticity. Neuroplasticity can modify the functions of the CNS including the brain and spinal cord, thereby providing opportunities for improving the limited ability of the CNS to recover from functional deficits. Axonal sprouting of survived neurons, new synapse formation, and factors produced by neurons and glia help reestablish the neural networks and functions. For example, at the cellular level, axonal sprouting, and dendritic arborization appear in injured areas. Peri-injury area and the repaired area of the injured tissue are getting attention for processes through which functional recovery occurs. In these areas, growth-promoting factors and growth-inhibitory proteins are released after injury. Genes which are related to survival, repair, and plasticity are also activated following injury. Enhanced electrical and chemical activities are expected to promote axonal sprouting, contributing to functional recovery. Indeed, axonal outgrowth after injury may be dependent on activities of neural circuits. Otherwise, silent synapses, pathways, or circuits may be activated.

Changes in the central neural circuits which are related to injured neurons play a crucial role not only in functional deficits but also in functional recovery. Furthermore, a variety of strategies including external interventions to manipulate neuroplasticity may be beneficial to improve functional recovery.

In this special issue, we focus on postinjury neural plasticity in central neural networks. Papers covering a wide spectrum of studies related to neural injury are included. The scientific reports updated results of both basic experimental studies in animals and clinical studies in humans. The original research articles as well as a review cover the field of neuroplastic changes in the CNS after injury and functional recovery.

Behavioral hypersensitivity may be related to biochemical alterations in the spinal cord. H. Y. Kim et al. show that elevated reactive oxygen species (ROS) in the spinal cord sensitized dorsal horn neurons and produced hyperalgesia in normal rats. M. C. Lee et al. report the relationship between the alteration of spinal GABAergic inhibition and mechanical hypersensitivity following unilateral chronic compression of dorsal root ganglion in rats.

Peripheral nerve injury can induce plastic changes in the spinal cord and the brain. In this regard, using an animal model of peripheral nerve injury, R. Won and B. H. Lee show that contralateral metabolic activation to nerve injury is related to behavioral crossed-withdrawal reflex in rats. Furthermore, J. Han et al. report that PKM zeta signaling in the insular cortex of the brain is involved in the modulation of neuropathic pain.

In relation to spinal cord injury, B. Azam et al. report experimental data suggesting that plastic changes of the 5-HT system mediated by aromatic L-amino acid decarboxylase cells might happen primarily in the subchronic phase and its function could be compensated by plastic changes of other modulation systems in longer chronic phase.

Stem cell therapy may be helpful to improve functional recovery after spinal cord injury. Experimentally, J. B. Iorgulescu et al. show that the combination of putrescine with Schwann cells improves functional recovery in rats with spinal cord injury. Clinically, J. C. Shin et al. report that the transplantation of human neural stem/progenitor cells (hNSPCs) into the injured cervical spinal cord is safe and provides modest neurological benefit up to 1 year after transplants.

Brain injury is much more dynamic and multifactorial compared to peripheral or spinal injury. In relation to functional recovery after brain injury, K. J. Cho et al. report that statins may affect not only the outcome of stroke by the recovery of noradrenergic (NA) neuronal circuitry but also the NA circuit's function after transient focal cerebral ischemia in mice. J. Song et al. provide experimental data suggesting that PKA inhibitor H89 may contribute to functional recovery after ischemic stroke by regulating neuronal death and proteins related to synaptic plasticity in mice. Clinically, J. P. Mäkelä et al. report covarying excitability measured with navigated transcranial magnetic stimulation and magnetoencephalography in the lesioned and nonlesioned hemispheres of stroke patients. As a systematic review, E. Wogensen et al. present a topic related to the effects of exercise on cognitive recovery after acquired brain injury in animal models.

This special issue compiles the state-of-the-art studies on central neuroplasticity after neural injury. We hope that this special issue will contribute to a better understanding of underlying mechanisms of neuroplasticity following neural injury and to development of novel neuromodulation strategies for functional recovery after injury.

